# Population intervention models of racial ethnic disparities in cognitive outcomes from cardiometabolic risk factors – HABS-HD

**DOI:** 10.1186/s13195-025-01866-9

**Published:** 2025-10-01

**Authors:** Cellas A. Hayes, Lubnaa Abdullah, Joshua Gills, Michelle C. Odden, The Health and Aging Brain Study (HABS-HD) Study Team

**Affiliations:** 1 Department of Epidemiology and Population Health, Stanford University School of Medicine, 1701 Page Mill Road, Palo Alto, CA 94304 USA; 2 Institute for Translational Research, University of North Texas Health Science Center, Fort Worth, TX USA; 3 Department of Family Medicine, University of North Texas Health Science Center, Fort Worth, TX USA; 4 Department of Psychiatry, Healthy Brain Aging Sleep Center, NYU Grossman School of Medicine, New York, NY USA; 5 Department of Population Health, Center for Healthful Behavior Change, Institute of Excellence in Health Equity, NYU Grossman School of Medicine, New York, NY USA

**Keywords:** Cardiometabolic risk factors, Hypertension, Diabetes, Apolipoprotein E, Neuropsychological testing, Cognition, Health disparities

## Abstract

**Background:**

Alzheimer’s disease and related dementias are major public health challenges, with the apolipoprotein (*APOE*) ε4 allele being a significant genetic risk factor. Cardiometabolic risk factors such as diabetes, hypertension, dyslipidemia, obesity, and tobacco use are also linked to cognitive impairment. The objective of this study was to (1) characterize both independent and interactive associations of racial/ethnic group (Non-Hispanic White (NHW), Non-Hispanic Black (NHB) and Hispanic), APOE ε4 genotype, and multiple cardiometabolic risk factors with performance across four cognitive domains. Secondarily, we aimed to quantify the hypothetical population-level cognitive gains that could result from eliminating each modifiable risk factor within each racial/ethnic group.

**Methods:**

We analyzed baseline data from 3,833 HABS-HD participants (1,348 NHW; 1,065 NHB; 1,420 Hispanic; mean age 65.3 ± 8.6 years; 62.0% female). APOE genotype, consensus-determined cardiometabolic status, and harmonized cognitive domain scores (episodic memory, executive function, processing speed, language) were obtained. Multivariable linear regressions assessed independent effects of race/ethnicity, APOE ε4 carriage, and each cardiometabolic factor on domain-specific z-scores, adjusting for age, sex, and education (Bonferroni-corrected). Interaction terms tested effect modification by race/ethnicity. Counterfactual population intervention models estimated the mean cognitive gain from hypothetically eliminating each modifiable risk factor within each racial/ethnic group.

**Results:**

NHB Hispanic, and NHW participants prevalences were APOE ε4 (32.2%, 27.4%, 17.6%), diabetes (26.1%, 35.0%, 13.9%), hypertension (79.0%, 63.6%, 58.2%), obesity (56.8%, 50.3%, 38.5%), and tobacco dependence (12.9%, 7.5%, 3.9%). In adjusted models, NHB and Hispanic ethnicity, APOE ε4, diabetes, hypertension, and tobacco dependence each independently predicted lower performance across all four cognitive domains (adjusted *p* < .001), whereas obesity showed domain-specific positive associations. No race × risk-factor interactions remained significant after correction. In intervention models, hypothetically eliminating diabetes and hypertension yielded the largest predicted improvements, especially in executive function and language, with the greatest gains projected among NHB and Hispanic racial ethnic group.

**Conclusions:**

Cardiometabolic health markedly contributes to racial ethnic differences in cognitive aging beyond *APOE* ε4 effects. Population-level interventions targeting diabetes and hypertension could narrow NHB and Hispanic cognitive deficits, informing precision public-health strategies for dementia prevention.

**Supplementary Information:**

The online version contains supplementary material available at 10.1186/s13195-025-01866-9.

## Introduction

Alzheimer’s disease (AD) and related dementias (ADRD) are major public health concerns, with cognitive impairment affecting millions worldwide [1]. The apolipoprotein (*APOE*) ε4 allele is one of the most significant genetic risk factors for AD [2]. *APOE* ε4 carriers experience higher rates of cognitive decline in early and late onset AD [3].

The association between APOE ε4 and cognitive impairment appears to be less pronounced in Black/African American populations compared to Non-Hispanic White (NHW) populations, highlighting the need for research that considers the influence of other risk factors for cognitive deficits including cardiometabolic risk factors that could drive vascular contribution to cognitive impairment [4]. While large cohorts such as the Atherosclerosis Risk in Communities and the Healthy Aging in Neighborhoods of Diversity across the Life Span have documented race-specific *APOE* ε4 effects on cognition in NHW and NH-Black (NHB) individuals, no prior study has simultaneously included Hispanic participants in a single community-based sample or applied counterfactual population intervention models to quantify how cardiometabolic conditions may explain those differences [4, 5].

Cardiometabolic conditions such as diabetes, hypertension, dyslipidemia, obesity, and tobacco use are associated with cognitive decline and increased dementia risk [6, 7]. Cardiometabolic health plays a critical role in brain aging, influencing vascular health, inflammatory processes, and metabolic regulation, all of which impact cognitive function [7]. For example, diabetes and hypertension have been consistently linked with poorer executive function and processing speed [8, 9], while obesity and dyslipidemia are associated with memory impairments [10, 11]. These health conditions are particularly prevalent in Black populations, potentially compounding risk factors for cognitive decline and influencing the interplay between genetics and other risk factors especially in mid-life [12–14].

Despite the well-documented link between *APOE* ε4 and cognitive decline, the interactions between this genetic risk factor and cardiometabolic health conditions across population prevalence racial ethnic backgrounds remain poorly understood [15]. In addition, previous research has assessed *APOE* ε4 and single cardiometabolic exposures in predominantly NHW with some comparison with NHB participants but often excluding Hispanic participants [4, 5]. A gap is the lack of translating those associations into hypothetical, population-level cognitive gains from risk factor elimination. Our study fills this gap by leveraging the Healthy Aging Brain Study-Health Disparities (HABS-HD) community cohort (NHW, NHB, and Hispanic participants) and using population intervention models to estimate the potential cognitive benefit of eliminating diabetes, hypertension, dyslipidemia, obesity, and tobacco dependence across all three groups across multiple cognitive domains.

The objective of this study was to [1] characterize both independent and interactive associations of racial/ethnic group (NHW, NHB, and Hispanic), APOE ε4 genotype, and cardiometabolic risk factors with performance across four cognitive domains, and [2] quantify the population intervention models, the hypothetical, population-level cognitive gains that could result from eliminating each modifiable risk factor within each racial/ethnic stratum. All analyses were conducted in the community-based HABS-HD cohort.

## Methods

### Study design

The HABS-HD cohort formerly known as Health & Aging Brain Among Latino Elders (HABLE) is a present longitudinal research study at the Institute for Translational Research in the University of North Texas Health Science Center [16]. HABS-HD is the largest comprehensive study of AD that includes NHW, NHB, and Hispanic participants [16]. As the first-ever large-scale study of Amyloid/Tau/Neurodegeneration framework and being a community based cohort, the HABS-HD study was designed to not only understand but help eliminate health disparities among populations that were not previously included and targeted in observational and clinical research studies [16]. The participants enrolled in HABS-HD complete comprehensive background/demographic questionnaires, comprehensive neuropsychological testing, clinical fluid laboratory assessments including blood draws, brain magnetic resonance imaging scans, positron emission tomography scans (amyloid and tau), and functional exams [16]. HABS-HD participant data are available upon request for research. All protocols and participants written informed consent was obtained and approved by the University of North Texas Health Science Center Institutional Review Board and follows the declaration of Helsinki guidelines.

### Data request and inclusion criteria

The full criteria to be a participant for HABS-HD has been described elsewhere [16]. For this study, baseline data were requested and received for all HABS-HD participants when data release 6 became available. Participants needed to have relevant demographic data including age, sex, and education, self-identify as NHW, NHB, or Hispanic, *APOE* genotype, self or clinical consensus indicator of cardiometabolic risk factors (diabetes, hypertension, dyslipidemia, obesity interpreted from body mass index (BMI), and tobacco dependence), and neuropsychological testing for cognition.

### APOE genotype

*APOE* ε4 genotyping was conducted using TaqMan Genotyping Kits for SNPs rs429158 and rs7412, in combination with the TaqMan GTXpress Master Mix from ThermoFisher Scientific [16, 17]. Amplification and detection of the target sequences were carried out on the Applied Biosystems 7500 Real-Time PCR System [16, 17]. Genotypes were determined based on allele amplification patterns at the two SNPs as follows: ε2/ε2 (T,T); ε2/ε3 (T,CT); ε2/ε4 (CT,CT); ε3/ε3 (T/C); ε3/ε4 (CT,C); and ε4/ε4 (C,C) [16, 17]. Each run included positive controls (samples with pre-determined *APOE* genotypes verified through independent methods) and negative controls [16, 17]. The distribution of *APOE* genotypes was verified to adhere to Hardy–Weinberg equilibrium [16, 17].

For analytical purposes, a binary variable distinguishing individual with at least one ε4 allele (ε4 carriers) from those without any ε4 allele (non-carriers) were utilized. This approach simplifies the analysis by grouping genotypes into two clinically relevant categories: those with increased risk of AD and cognitive impairment (ε4 carriers) and those without this genetic risk factor (non-ε4 carriers) although there is a dose-dependent relationship for *APOE* [18, 19]. This binary categorization enables more robust statistical power and interpretability, especially when assessing the association of *APOE* ε4 and its interactive effects with cardiometabolic factors. In addition, we could not perform dose-dependent analyses in this study due to the low prevalence of ε4/ε4 carriers.

### Cardiometabolic risk factors

The diagnoses of hypertension, diabetes, dyslipidemia, obesity, and tobacco dependence were determined through consensus review. Hypertension was defined by a self-reported medical diagnosis, use of antihypertensive medications, and/or an average of two blood pressure readings exceeding 140/90 mmHg. Diabetes was identified through a self-reported medical diagnosis, current use of insulin or oral hypoglycemic medications, and/or an HbA1c level greater than 6.5%. Dyslipidemia was characterized by a self-reported diagnosis of elevated cholesterol or triglycerides, use of lipid-lowering medications, total cholesterol levels above 200 mg/dL, and/or triglycerides exceeding 150 mg/dL. Obesity was defined by a body mass index (BMI) of 30.0 or higher. These risk factors have been utilized in previous HABS-HD studies [20, 21].

### Neuropsychological assessment

The details of the neuropsychological assessment for HABS-HD has been described previously [16]. Briefly, the following tests were conducted: Mini Mental Status Exam (MMSE) [22], Wechsler Memory Scale- Third Edition (WMS-III) Digit Span and Logical Memory [23], Digit Symbol Substitution, Trail Making Test Parts A and B [24], Spanish–English Verbal Learning Test (SEVLT) [25], Animal Naming (semantic fluency) [25], FAS (phonemic fluency) [26].

### Cognitive outcomes

The neuropsychological cognitive domains have been described previously in the HABS-HD data [16]. In this study, the episodic memory composite score was constructed by averaging the z-scores of four episodic memory tasks: SEVLT Delayed Recall, SEVLT Immediate Recall, Logical Memory 1, and Logical Memory 2. Executive function was represented by a composite score calculated as the mean of z-scores from the Digit Span and Digit Symbol Substitution tests. Processing speed was indexed by combining z-scores from the Trails A and Trails B tests and inverted to reflect the same direction for analytical outcomes. Lastly, language ability was assessed by averaging the z-scores from the Animal Naming and FAS Verbal Fluency tests. Overall, to create these cognitive domain scores, we standardized the individual cognitive measures into z-scores and then computed composite scores by averaging these z-scores following similar HABS-HD methodology [27].

- Episodic memory tasks: SEVLT Delayed Recall, SEVLT Immediate Recall, Logical Memory 1, and Logical Memory 2.
- Executive function: Digit Span and Digit Symbol Substitution tests.
- Processing speed: Trails A and Trails B tests.
- Language: Animal Naming and FAS Verbal Fluency tests.

### Statistical analyses

Participant characteristics were summarized stratified by racial ethnicity (NHW, NHB, and Hispanic). Continuous variables (age, education, CDR Sum of Boxes, BMI, memory composite, executive-function composite, processing-speed composite, language composite) are presented as mean (standard deviation (SD)). Categorical variables (sex, *APOE* ε4 carriership, *APOE* genotype category, diabetes, hypertension, dyslipidemia, obesity [BMI ≥ 30], tobacco dependence, cardiovascular disease history, and cognitive status) are reported as counts and percentages. Between-group differences for continuous measures were evaluated using one-way ANOVA, with Tukey’s Honest Significant Difference (HSD) test for pairwise comparisons. Differences in categorical measures were assessed with Pearson’s chi-square tests; when expected counts were < 5, Fisher’s exact test with Monte Carlo simulation (10,000 replicates) was used.

### Multivariable linear regression models

For each of the four harmonized cognitive domains (memory, executive function, processing speed, and language), we fit a fully adjusted model. The model included age, education, sex, *APOE* ε4, racial ethnicity, diabetes, hypertension, dyslipidemia, obesity status, and tobacco dependence. For all four models, we corrected for multiple comparisons using the Bonferroni method. The adjusted level of significant accounted for 48 tests (adjusted *p* = 0.00104). To assess the interactions between racial ethnicity (NHW, NHB, and Hispanic) with *APOE* ε4 and racial ethnicity (NHW, NHB, and Hispanic) with the 5 cardiometabolic risk factor (diabetes, hypertension, dyslipidemia, obesity status, and tobacco dependence) on cognition, we used a single regression model as a parsimonious approach [28–30]. The model example is: cognition ~ age + education + sex + *APOE* ε4 * racial ethnicity + racial ethnicity * diabetes + racial ethnicity * hypertension + racial ethnicity * dyslipidemia + racial ethnicity * obesity + racial ethnicity * tobacco dependence. We also applied a Bonferroni correction accounting for 96 tests (adjusted *p*-value = 0.000521). Each model reported estimated coefficients (β) and 95% confidence intervals (CI) for both main and interaction effects. In addition, we report both unadjusted and Bonferroni‐adjusted *p*-values.

### Population intervention models

To evaluate the impact of eliminating specific cardiometabolic risk factors on cognitive performance, we implemented counterfactual-based population intervention models (PIM) using the g-computation substitution estimator. This method, similar to a population attributable fraction, models the hypothetical change in outcome if the exposure were eliminated [31–33]. We fit a pooled linear regression to the full sample adjusted for age, sex, education, *APOE* ε4 status, racial ethnicity, diabetes, hypertension, dyslipidemia, obesity, and tobacco dependence and domain‐specific cognitive scores. We quantified three counterfactual scenarios: (1) all participants exposed, (2) none exposed, and (3) observed exposure. Predictions were stratified by self-reported racial ethnicity (NHW, NHB, and Hispanic) to obtain group-specific estimates. We defined PIM as the mean difference between the “none exposed” and “observed” scenarios, representing the hypothetical effect of eliminating the exposure. All reported PIM estimates were computed separately for each racial ethnic group and across four harmonized domains (episodic memory, executive function, processing speed, and language), with 95% CI derived from 1,000 bootstrap resamples. Positive PIM values indicate potential cognitive benefit from eliminating the risk factor, whereas negative values indicate potential cognitive harm. We report PIM only for those risk factors that demonstrated significant main effects in the models without interactions.

All statistical analyses were conducted using R version 4.2.3.

## Results

### Demographics and clinical characteristics

Participant characteristics varied significantly by racial ethnicity (all *p* < 0.001, Table 1). NHB (mean age 63.4 years) and Hispanics (63.3 years) were both about 5.5 years younger than NHW (68.8 years). Educational attainment followed a similar pattern: NHW averaged 15.5 years of schooling, NHB 14.7 years, and Hispanics 10.3 years. BMI was highest in NHW (32.2 kg/m2), intermediate in Hispanics (31.0 kg/m2), and lowest in NHW (29.1 kg/m2). Cardiometabolic risks were also unevenly distributed: diabetes prevalence was 13.9% in NHW, 26.1% in NHB, and 35.0% in Hispanics; hypertension affected 58.2%, 79.0%, and 63.6% of each group, respectively; and dyslipidemia was most common in Hispanics (73.4%) versus 68.4% of NHW and 62.4% of NHB. Obesity and tobacco dependence likewise were significantly more prevalent in NHB and Hispanics compared to NHW (all *p* < 0.001). Cognitive scores mirrored these health disparities: NHW performed highest, NHB intermediate, and Hispanics lowest across memory, executive function, processing speed, and language (all *p* < 0.001). Similarly, 79.9% of NHW, 64.4% of NHB, and 71.9% of Hispanics were classified as cognitively normal (χ2 *p* < 0.001).

**Table 1:** Demographic, cardiometabolic, and cognitive characteristics of participants stratified by racial ethnicity

	Overall (*N* = 3833)	Non-Hispanic White (*N* = 1348)	Non-Hispanic Black (*N* = 1065)	Hispanic (*N* = 1420)	*p*-value
Age (years), Mean (SD)	65.3 (8.6)	68.8 (8.7)	63.4 (7.8)	63.3 (8.1)	< 0.001
Education (years), Mean (SD)	13.4 (4.2)	15.5 (2.6)	14.7 (2.6)	10.3 (4.6)	< 0.001
Female, n (%)	2376 (62.0)	767 (56.9)	672 (63.1)	937 (66.0)	< 0.001
APOE ε4, n (%)	962 (25.1)	369 (27.4)	343 (32.2)	250 (17.6)	< 0.001
Missing, n (%)	494 (12.9)	114 (8.5)	235 (22.1)	145 (10.2)	
APOE Genotype, n (%)					< 0.001
ε2ε2	15 (0.4)	5 (0.4)	9 (0.8)	1 (0.1)	
ε2ε3	331 (8.6)	150 (11.1)	101 (9.5)	80 (5.6)	
ε2ε4	76 (2.0)	25 (1.9)	41 (3.8)	10 (0.7)	
ε3ε3	2031 (53.0)	710 (52.7)	377 (35.4)	944 (66.5)	
ε3ε4	792 (20.7)	314 (23.3)	253 (23.8)	225 (15.8)	
ε4ε4	94 (2.5)	30 (2.2)	49 (4.6)	15 (1.1)	
Missing, n (%)	494 (12.9)	114 (8.5)	235 (22.1)	145 (10.2)	
Diabetes, n (%)	963 (25.1)	188 (13.9)	278 (26.1)	497 (35.0)	< 0.001
Hypertension, n (%)	2528 (66.0)	784 (58.2)	841 (79.0)	903 (63.6)	< 0.001
Missing, n (%)	1 (0.0)	0 (0)	1 (0.1)	0 (0)	
Dyslipidemia, n (%)	2629 (68.6)	922 (68.4)	665 (62.4)	1042 (73.4)	< 0.001
Body Mass Index kg/m2, Mean (SD)	30.7 (6.7)	29.1 (6.2)	32.2 (7.7)	31.0 (6.1)	< 0.001
Missing, n (%)	24 (0.6)	9 (0.7)	9 (0.8)	6 (0.4)	
Obesity, n (%)	1838 (48.0)	519 (38.5)	605 (56.8)	714 (50.3)	< 0.001
Missing, n (%)	24 (0.6)	9 (0.7)	9 (0.8)	6 (0.4)	
Tobacco Dependency, n (%)	296 (7.7)	52 (3.9)	137 (12.9)	107 (7.5)	< 0.001
CDR Sum of Boxes, Mean (SD)	0.5 (1.5)	0.4 (1.3)	0.7 (1.6)	0.6 (1.5)	< 0.001
Missing, n (%)	1 (0.0)	0 (0)	1 (0.1)	0 (0)	
Episodic Memory, Mean (SD)	−0.0 (0.9)	0.3 (0.9)	−0.1 (0.9)	−0.2 (0.8)	< 0.001
Missing, n (%)	4 (0.1)	1 (0.1)	0 (0)	3 (0.2)	
Executive Function, Mean (SD)	−0.0 (0.9)	0.4 (0.8)	0.1 (0.8)	−0.5 (0.8)	< 0.001
Missing, n (%)	1 (0.0)	0 (0)	0 (0)	1 (0.1)	
Processing Speed, Mean (SD)	−0.0 (1.0)	0.3 (0.7)	0.0 (0.9)	−0.4 (1.1)	< 0.001
Missing, n (%)	19 (0.5)	5 (0.4)	4 (0.4)	10 (0.7)	
Language, Mean (SD)	−0.0 (0.9)	0.3 (0.9)	0.03 (0.9)	−0.3 (0.8)	< 0.001
History of Cardiovascular Disease, n (5)	278 (7.3)	133 (9.9)	74 (6.9)	71 (5.0)	< 0.001
Cognitive Status, n (%)					< 0.001
Normal	2784 (72.6)	1077 (79.9)	686 (64.4)	1021 (71.9)	
Mild Cognitive Impairment	801 (20.9)	191 (14.2)	306 (28.7)	304 (21.4)	
Dementia	236 (6.2)	79 (5.9)	68 (6.4)	89 (6.3)	
Unknown	12 (0.3)	1 (0.1)	5 (0.5)	6 (0.4)	

This table presents demographic, cardiometabolic, and harmonized cognitive characteristics of study participants, stratified by Non-Hispanic White, Non-Hispanic Black, and Hispanic racial ethnicities. Continuous variables are displayed as mean (standard deviation), and categorical variables are shown as counts and percentages.. Between-group differences for continuous measures were evaluated using one-way ANOVA, with Tukey’s Honest Significant Difference (HSD) test for pairwise comparisons. Differences in categorical measures were assessed with Pearson’s chi-square tests; when any cell’s expected count was < 5, Fisher’s exact test with Monte Carlo simulation (10,000 replicates) was used. Abbreviations: APOE apolipoprotein E, SD standard deviation

### Racial/ethnic group differences

Compared to NHW, NHB exhibited significantly lower performance across all cognitive domains: episodic memory (β = –0.51; 95% CI: –0.59 to –0.44; adjusted *p* < 0.001), executive function (β = –0.38; 95% CI: –0.44 to –0.32; adjusted *p* < 0.001), processing speed (β = –0.35; 95% CI: –0.42 to –0.28; adjusted *p* < 0.001), and language (β = –0.27; 95% CI: –0.35 to –0.20; adjusted *p* < 0.001) (Table 2). Hispanic participants also scored lower than NHW on each domain: memory (β = –0.30; 95% CI: –0.38 to –0.23; adjusted *p* < 0.001), executive function (β = –0.50; 95% CI: –0.56 to –0.44; adjusted *p* < 0.001), processing speed (β = –0.25; 95% CI: –0.32 to –0.17; adjusted *p* < 0.001), and language (β = –0.22; 95% CI: –0.29 to –0.14; adjusted *p* < 0.001).

**Table 2:** The main effect associations of racial ethnicity, *APOE* ε4, and cardiometabolic conditions on cross-sectional harmonized cognitive domain scores in the overall sample

	Episodic Memory (*N* = 3,311)			Executive Function (*N* = 3,314)			Processing Speed (*N* = 3,300)			Language (*N* = 3,315)		
*Predictors*	β (95% CI)	*Unadj. p*	*Adj.p*	β (95% CI)	*Unadj. p*	*Adj.p*	β (95% CI)	*Unadj. p*	*Adj.p*	β (95% CI)	*Unadj. p*	*Adj.p*
*APOE* ε4	** −0.14 (−0.20 – −0.08)**	** < 0.01**	** < 0.01**	** −0.05 (−0.10 – −0.00)**	**0.04**	0.49	** −0.06 (−0.12 – −0.00)**	**0.04**	0.42	** −0.07 (−0.12 – −0.01)**	**0.03**	0.31
NHB vs NHW	** −0.51 (−0.59 – −0.44)**	** < 0.01**	** < 0.01**	** −0.38 (−0.44 – −0.32)**	** < 0.01**	** < 0.01**	** −0.35 (−0.42 – −0.28)**	** < 0.01**	** < 0.01**	** −0.27 (−0.35 – −0.20)**	** < 0.01**	** < 0.01**
Hispanic vs NHW	** −0.30 (−0.38 – −0.23)**	** < 0.01**	** < 0.01**	** −0.50 (−0.56 – −0.44)**	** < 0.01**	** < 0.01**	** −0.25 (−0.32 – −0.17)**	** < 0.01**	** < 0.01**	** −0.22 (−0.29 – −0.14)**	** < 0.01**	** < 0.01**
Diabetes	−0.01 (−0.07 – 0.06)	0.84	1.00	−0.04 (−0.10 – 0.01)	0.11	1.00	** −0.09 (−0.16 – −0.03)**	** < 0.01**	**0.04**	** −0.08 (−0.14 – −0.02)**	**0.01**	0.12
Hypertension	−0.02 (−0.08 – 0.04)	0.47	1.00	** −0.05 (−0.10 – −0.01)**	**0.03**	0.35	−0.03 (−0.09 – 0.02)	0.24	1.00	** −0.07 (−0.13 – −0.02)**	**0.01**	0.16
Dyslipidemia	0.02 (−0.04 – 0.07)	0.56	1.00	−0.01 (−0.06 – 0.04)	0.75	1.00	0.03 (−0.03 – 0.08)	0.34	1.00	0.02 (−0.04 – 0.08)	0.50	1.00
Obesity	**0.09 (0.04–0.15)**	** < 0.01**	**0.01**	−0.00 (−0.05 – 0.04)	0.94	1.00	**0.08 (0.02–0.13)**	**0.01**	0.07	−0.00 (−0.05 – 0.05)	1.00	1.00
Tobacco Dependence	** −0.21 (−0.32 – −0.11)**	** < 0.01**	** < 0.01**	** −0.12 (−0.21 – −0.03)**	**0.01**	0.08	−0.08 (−0.18 – 0.02)	0.13	1.00	** −0.13 (−0.23 – −0.03)**	**0.01**	0.12

Linear regression results for four harmonized cognitive domains—episodic memory (*N* = 3,311), executive function (*N* = 3,314), processing speed (*N* = 3,300), and language (*N* = 3,315)—showing the main effects of racial ethnic group (Non-Hispanic Black vs. Non-Hispanic White; Hispanic vs. Non-Hispanic White), APOE ε4 carriership (≥ 1 ε4 allele vs. none), and five cardiometabolic conditions (diabetes, hypertension, dyslipidemia, obesity, tobacco dependence), adjusted for age (years), education (years), and sex (female vs. male). The unstandardized beta coefficient (β) with its 95% confidence interval (CI), the unadjusted *p*-value, and the Bonferroni-adjusted *p*-value (α* = 0.05/48 = 0.00104, to account for 48 tests across the four models) are shown. Bold values indicate significance for unadjusted and adjusted *p*-values

### APOE ε4 carriership

Carrying at least one *APOE* ε4 allele was associated with poorer episodic memory (β = –0.14; 95% CI –0.20 to –0.08; adjusted *p* < 0.001) (Table 2). Although ε4 carriership nominally predicted lower scores in executive function (β = –0.05; 95% CI: –0.10 to –0.00; unadjusted *p* = 0.04), processing speed (β = –0.06; –0.12 to –0.00; unadjusted *p* = 0.04), and language (β = –0.07; –0.12 to –0.01; unadjusted *p* = 0.03), these associations did not remain significant after Bonferroni correction (adjusted *p* > 0.3).

### Cardiometabolic risk factors

**Fig. 1:**
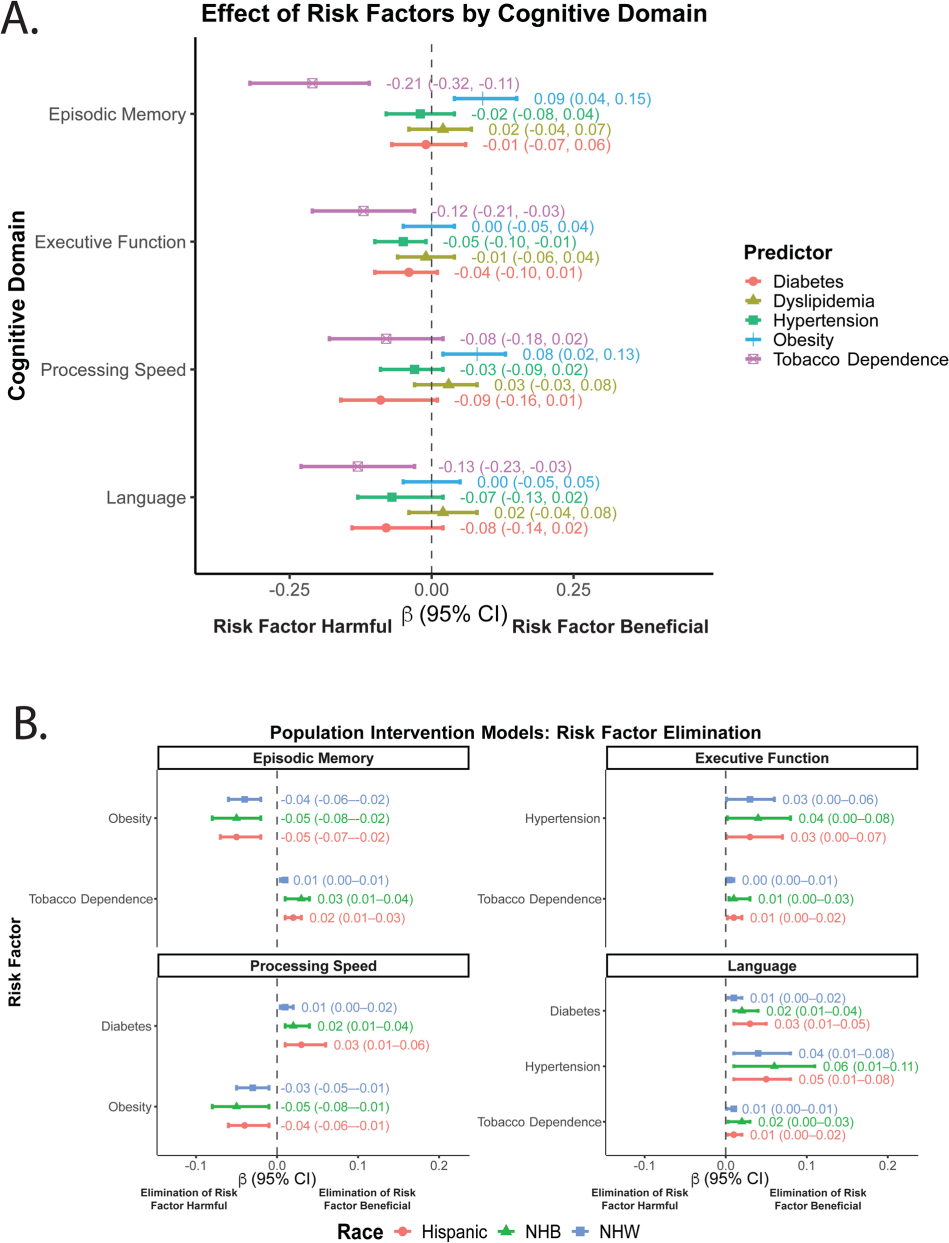
Associations between hypothetical removal of cardiometabolic risk factors on cognitive performance. Panel A: Forest plot displaying the independent associations (β coefficients and 95% confidence intervals) between five cardiometabolic risk factors—diabetes, hypertension, dyslipidemia, obesity, and tobacco use—and harmonized cognitive domain scores in the overall analytic sample. Cognitive domains include episodic memory, executive function, processing speed, and language. All five risk factors were included simultaneously in each model. Models were adjusted for age, sex, education (years), apolipoprotein ε4 allele status, and racial ethnicity. Domains are distinguished by color and shape: episodic memory (red circles), executive function (green triangles), language (blue squares), and processing speed (purple crosses). Lines extending from points represent 95% confidence intervals for each estimate. Risk factors being harmful and beneficial are indicated below the x-axis estimates. Panel B: Domain-specific Forest plots showing racial-ethnic-stratified estimates from counterfactual population intervention models, which estimate the potential difference in cognitive performance if participants had not been exposed (non-treated) to each cardiometabolic risk factor, compared to their observed exposure status. Results are stratified by Non-Hispanic White (NHW; blue) and Non-Hispanic Black (NHB; green) and Hispanic (red) participants. Positive estimates indicate improved cognitive performance under the non-treatment scenario, while negative estimates suggest reduced performance. Each cognitive domain is shown in a separate panel for clarity. Hypothetical elimination of risk factors being harmful and beneficial are indicated below the x-axis estimates. Lines extending from points denote 95% confidence intervals. Abbreviations: β = beta coefficient; CI = confidence interval; PIM = population intervention model; NHW = Non-Hispanic White; NHB = Non-Hispanic Black

Among the cardiometabolic variables, obesity was linked to better episodic memory (β = 0.09; 95% CI: 0.04 to 0.15; adjusted *p* = 0.01), while tobacco dependence was associated with worse memory (β = –0.21; 95% CI: –0.32 to –0.11; adjusted *p* < 0.001) (Table 2, Fig. 1A). Diabetes demonstrated a modest negative effect on processing speed (β = –0.09; 95% CI: –0.16 to –0.03; adjusted *p* = 0.04), and hypertension was related to lower language performance (β = –0.07; 95% CI: –0.13 to –0.02; adjusted *p* = 0.01).

### Interaction associations

After correcting for multiple comparisons (Bonferroni-adjusted α* = 0.00052), none of the *APOE* ε4 × racial ethnicity or racial ethnicity × cardiometabolic risk interactions remained statistically significant across any cognitive domain (Table 3). Nominally, we observed a trend toward a stronger negative effect of *APOE* ε4 on episodic memory in NHB versus NHW (interaction β = 0.13; 95% CI: –0.01 to 0.27; unadjusted *p* = 0.07), and the diabetes × Hispanic interaction hinted at slightly better memory among Hispanics with diabetes (β = 0.16; 95% CI: 0.01 to 0.32; unadjusted *p* = 0.04), but neither survived adjustment (adjusted *p* > 0.90). Similarly, hypertension appeared to have a more deleterious association with executive function in NHB (β = –0.13; 95% CI: –0.20 to –0.05; unadjusted *p* < 0.01) and language in Hispanics (β = 0.11; 95% CI: 0.01 to 0.26; unadjusted *p* = 0.04), yet these interaction effects did not meet the stringent corrected threshold.

**Table 3:** Interaction effects of racial ethnic group with APOE ε4 carriership and cardiometabolic risk factors on cross‐sectional harmonized cognitive domain scores

	Episodic Memory (*N* = 3,311)			Executive Function (*N* = 3,314)			Processing Speed (*N* = 3,300)			Language (*N* = 3,315)		
*Predictors*	β (95% CI)	*Unadj. p*	*Adj. p*	β (95% CI)	*Unadj. p*	*Adj. p*	β (95% CI)	*Unadj. p*	*Adj. p*	β (95% CI)	*Unadj. p*	*Adj. p*
*APOE* ε4	** −0.20 (−0.29 – −0.11)**	** < 0.01**	** < 0.01**	** −0.08 (−0.16 – −0.00)**	**0.04**	0.89	−0.08 (−0.18 – 0.01)	0.08	1.00	** −0.10 (−0.20 – −0.01)**	**0.03**	0.63
NHB vs NHW	** −0.65 (−0.82 – −0.47)**	** < 0.01**	** < 0.01**	** −0.45 (−0.59 – −0.30)**	** < 0.01**	** < 0.01**	** −0.38 (−0.55 – −0.20)**	** < 0.01**	** < 0.01**	** −0.32 (−0.49 – −0.14)**	** < 0.01**	**0.01**
Hispanic vs NHW	** −0.28 (−0.43 – −0.14)**	** < 0.01**	** < 0.01**	** −0.52 (−0.65 – −0.40)**	** < 0.01**	** < 0.01**	−0.10 (−0.25 – 0.05)	0.18	1.00	** −0.25 (−0.39 – −0.10)**	** < 0.01**	**0.02**
Diabetes	−0.09 (−0.21 – 0.04)	0.17	1.00	−0.03 (−0.14 – 0.08)	0.59	1.00	−0.09 (−0.21 – 0.04)	0.19	1.00	** −0.15 (−0.28 – −0.03)**	**0.02**	0.38
Hypertension	−0.03 (−0.12 – 0.06)	0.56	1.00	** −0.13 (−0.20 – −0.05)**	** < 0.01**	**0.03**	−0.04 (−0.13 – 0.05)	0.41	1.00	** −0.15 (−0.24 – −0.06)**	** < 0.01**	**0.02**
Dyslipidemia	0.00 (−0.09 – 0.10)	0.93	1.00	0.03 (−0.05 – 0.11)	0.44	1.00	**0.10 (0.01–0.19)**	**0.04**	0.91	0.06 (−0.03 – 0.15)	0.19	1.00
Obesity	**0.14 (0.05–0.23)**	** < 0.01**	0.08	−0.01 (−0.09 – 0.07)	0.79	1.00	0.09 (−0.00 – 0.18)	0.06	1.00	0.04 (−0.05 – 0.13)	0.34	1.00
Tobacco Dependence	−0.14 (−0.37 – 0.09)	0.22	1.00	0.03 (−0.16 – 0.22)	0.78	1.00	−0.06 (−0.29 – 0.17)	0.61	1.00	−0.13 (−0.36 – 0.10)	0.26	1.00
*APOE* ε4 x NHB	0.13 (−0.01 – 0.27)	0.07	1.00	0.03 (−0.09 – 0.15)	0.61	1.00	0.02 (−0.12 – 0.16)	0.78	1.00	0.03 (−0.11 – 0.17)	0.69	1.00
*APOE* ε4 x Hispanic	0.05 (−0.08 – 0.19)	0.44	1.00	0.06 (−0.06 – 0.18)	0.31	1.00	0.04 (−0.10 – 0.18)	0.55	1.00	0.09 (−0.05 – 0.23)	0.20	1.00
NHB x Diabetes	0.00 (−0.17 – 0.18)	0.96	1.00	−0.09 (−0.24 – 0.05)	0.22	1.00	−0.05 (−0.23 – 0.12)	0.56	1.00	0.02 (−0.16 – 0.19)	0.84	1.00
Hispanic x Diabetes	**0.16 (0.01–0.32)**	**0.04**	0.93	0.02 (−0.11 – 0.15)	0.75	1.00	0.02 (−0.14 – 0.17)	0.85	1.00	0.14 (−0.01 – 0.29)	0.08	1.00
NHB x Hypertension	0.07 (−0.08 – 0.23)	0.36	1.00	0.13 (−0.01 – 0.26)	0.06	1.00	0.03 (−0.13 – 0.19)	0.68	1.00	0.12 (−0.03 – 0.28)	0.13	1.00
Hispanic x Hypertension	−0.03 (−0.16 – 0.09)	0.62	1.00	**0.11 (0.00–0.22)**	**0.04**	1.00	−0.01 (−0.14 – 0.12)	0.87	1.00	**0.13 (0.01–0.26)**	**0.04**	0.95
NHB x Dyslipidemia	0.06 (−0.08 – 0.20)	0.42	1.00	−0.03 (−0.16 – 0.09)	0.58	1.00	−0.04 (−0.18 – 0.11)	0.61	1.00	−0.03 (−0.18 – 0.11)	0.66	1.00
Hispanic x Dyslipidemia	−0.01 (−0.14 – 0.12)	0.87	1.00	−0.08 (−0.19 – 0.03)	0.18	1.00	** −0.17 (−0.30 – −0.04)**	**0.01**	0.30	−0.09 (−0.23 – 0.04)	0.16	1.00
NHB x Obesity	0.00 (−0.13 – 0.14)	0.96	1.00	0.06 (−0.06 – 0.18)	0.30	1.00	0.06 (−0.08 – 0.20)	0.37	1.00	−0.04 (−0.17 – 0.10)	0.60	1.00
Hispanic x Obesity	−0.11 (−0.23 – 0.02)	0.09	1.00	−0.02 (−0.12 – 0.09)	0.74	1.00	−0.07 (−0.20 – 0.05)	0.24	1.00	−0.08 (−0.20 – 0.04)	0.19	1.00
NHB x Tobacco Dependence	−0.15 (−0.43 – 0.12)	0.28	1.00	** −0.24 (−0.47 – −0.01)**	**0.04**	1.00	−0.03 (−0.31 – 0.24)	0.82	1.00	−0.05 (−0.33 – 0.22)	0.70	1.00
Hispanic x Tobacco Dependence	−0.01 (−0.28 – 0.27)	0.97	1.00	−0.11 (−0.34 – 0.13)	0.37	1.00	0.00 (−0.28 – 0.28)	0.99	1.00	0.06 (−0.21 – 0.34)	0.66	1.00

Linear regression interaction models were fit separately for each cognitive domain—episodic memory (*N* = 3,311), executive function (*N* = 3,314), processing speed (*N* = 3,300), and language (*N* = 3,315)—adjusting for age (years), education (years), sex (female vs. male), APOE ε4 carriership (≥ 1 ε4 allele vs. none), racial ethnicity (Non-Hispanic White [reference], Non-Hispanic Black, Hispanic), and five cardiometabolic risk factors (diabetes, hypertension, dyslipidemia, obesity, tobacco dependence). Each model included two sets of interaction terms: APOE ε4 × racial ethnicity and racial ethnicity × each cardiometabolic risk factor. Shown are unstandardized beta coefficients (β) with 95% confidence intervals (CI), unadjusted *p*-values, and Bonferroni-adjusted *p*-values (α* = 0.05/96 = 0.00052 to account for all 96 tested parameters). Statistically significant adjusted *p*-values (< 0.05) are bolded

### Population intervention model effects from significant main effects

Population intervention model effects are depicted in Fig. 1B, Supplemental Table 1. In episodic memory, eliminating obesity (versus the observed obesity distribution) was associated with lower predicted scores across all groups, NHW: PIM = –0.04 (95% CI –0.06 to –0.02), NHB: –0.05 (–0.08 to –0.02), Hispanic: –0.05 (–0.07 to –0.02). By contrast, tobacco dependence showed a small positive PIM, NHW: 0.01 (0.004–0.01), NHB: 0.03 (0.01–0.04), Hispanic: 0.02 (0.01–0.03), suggesting minor memory benefits under a “no tobacco” scenario. For executive function, eliminating hypertension predicted modest gains across the racial ethnic groups, NHW: 0.03 (0.001–0.06), NHB: 0.04 (0.002–0.08), Hispanic: 0.03 (0.001–0.07). In addition, removing tobacco dependence resulted in smaller improvements, NHW: 0.004 (0.001–0.01), NHB: 0.01 (0.004–0.03), Hispanic: 0.01 (0.002–0.02). In processing speed, diabetes elimination predicted small improvements—NHW: 0.01 (0.004–0.02), NHB: 0.02 (0.01–0.04), Hispanic: 0.03 (0.01–0.06)—while obesity removal again suggested slight decrements (NHW: –0.03 [–0.05 to –0.01], NHB: –0.05 [–0.08 to –0.01], Hispanic: –0.04 [–0.06 to –0.01]). Finally, for language, both diabetes and hypertension elimination predicted improvements—diabetes: NHW: 0.01 (0.003–0.02), NHB: 0.02 (0.01–0.04), Hispanic: 0.03 (0.01–0.05); hypertension: NHW: 0.04 (0.01–0.08), NHB: 0.06 (0.01–0.11), Hispanic: 0.05 (0.01–0.08)—and tobacco elimination showed modest benefits—NHW: 0.01 (0.001–0.01), NHB: 0.02 (0.003–0.03), Hispanic: 0.01 (0.002–0.02).

## Discussion

In this analysis of older adults from the HABS-HD cohort, both NHB and Hispanic participants exhibited consistently lower scores than NHW participants across all four harmonized cognitive domains, episodic memory, executive function, processing speed, and language. After adjusting for age, education, and sex, carrying an APOE ε4 allele remained a robust predictor of poorer episodic memory, while its nominal associations with other domains did not survive correction for multiple comparisons. Among cardiometabolic factors, diabetes was linked specifically to slower processing speed and hypertension to poorer language performance, whereas tobacco dependence was associated with poorer cognition across all domains except processing speed. In contrast, obesity showed positive associations with memory and processing speed, respectively.

Although the effect of the risk factors did not differ by racial ethnicity, the prevalence of cardiometabolic risk factors was higher among NHB participants following previous trends [34]. To quantify the potential population-level benefits of eliminating these risk factors, we applied counterfactual population intervention models which are similar to the population attributable fraction[31–33]. Across nearly all domains, NHB participants stood to gain more from risk-factor elimination than NHW. In particular, removal of hypertension predicted larger improvements in executive function and language among NHB, and tobacco cessation yielded greater memory, executive function, and language benefits. With the removal of diabetes, Hispanic participants would be expected to have the greatest improvements in processing speed and language. Our findings are consistent with broader epidemiologic evidence showing that NHB populations experience higher prevalence, earlier onset, and greater severity of some cardiometabolic conditions like hypertension [34]. These PIM results underscore that, even in the absence of statistically significant interaction terms, disparities in risk-factor prevalence can translate into meaningful differences in potential cognitive gains across racial and ethnic groups.

Our observation of lower cognitive scores among NHB and Hispanic versus NHW older adults across multiple domains builds directly on Mehta and colleagues finding that Black participants in the Health ABC cohort scored substantially lower on global cognition and processing speed than White participants [35]. Mehta and colleagues showed that adjusting for literacy and education attenuated 86% of the Black–White gap, whereas demographic and psychosocial factors explained less variance in the model or less differences between race [35]. Our study extends this work by demonstrating that cardiometabolic risk factors contribute to these disparities despite NHB and NHW having similar years of education. Although educational attainment remains a powerful determinant of cognitive resilience and decline, our counterfactual PIM reveal that eliminating health-related risk factors could yield significant cognitive gains among NHB and Hispanic adults, owing to their higher prevalence of these conditions [36, 37].

*APOE* ε4 carriers in the ARIC cohort experienced earlier dementia onset, particularly among White participants, while our findings underscore the role of *APOE* ε4 with poorer episodic memory even after accounting for age, education, and sex [5]. Similarly, findings are presented with ε4 carriers having a 36–50% elevated risk of incident mild cognitive impairment and accelerated decline across memory and other domains [38]. Our findings are comparable with strongest effect of *APOE* ε4 being in the episodic memory domain while observing only nominal, non-significant associations with executive function, processing speed, and language after multiple-comparison correction, mirroring Barnes and colleagues who found ε4-driven semantic and working memory decline in White participants but not in Black participants [39].

Our domain-specific associations for cardiometabolic factors are aligned with previous findings which demonstrated that diabetes modestly increased AD risk and tripled stroke-related dementia risk, and these effects were higher in Black and Hispanic older adults [40]. In our cohort, diabetes was associated with slower processing speed composite, suggesting possible microvascular dysfunction [41], whereas hypertension impaired language performance designated exams, likely reflecting microvascular injury in alternative networks and regions [42–44]. Lower BMI predicted faster decline in semantic and episodic memory without race interactions [45]. We observed positive cross-sectional associations between obesity and both memory and processing speed. However, the estimates for the effects of elimination of obesity and dyslipidemia was paradoxically associated with worse processing speed performance, as indicated by negative PIM values, and elimination of obesity was associated with worse memory These counterintuitive findings may reflect the previous findings that lower BMI later in life is associated with lower bone mineral density and an increased risk for cognitive decline [46–48]. Our findings between obesity and cognition are also supported by results indicating that there is a linear association between increasing weight loss and clinical dementia rating stages in over 16,000 older adults [49]; declining BMI is associated with increases in dementia diagnoses 10 years prior [50]; and a higher BMI is associated with better cognition in mild cognitive impairment participants [51].

Also, the PIM derived estimates that eliminating hypertension would yield the largest cognitive gains, particularly in executive function and language among NHB adults, are aligned with the SPRINT MIND clinical trial demonstration that intensive systolic blood pressure lowering to < 120 mmHg significantly reduced incident mild cognitive impairment and the composite of MCI compared with a target of < 140 mm Hg [52]. Our counterfactual models suggest that, at the population level, even modest vascular risk reductions could translate into meaningful prevention of early cognitive decline across diverse racial and ethnic groups. In particular, the larger predicted benefit among NHB participants underscores how targeted intensive blood pressure management might help close racial disparities in late-life cognitive health.

Several limitations should be noted. First, because 72.6% of our sample was cognitively normal (20.9% MCI; 6.2% dementia), our findings may not fully generalize to individuals with more advanced impairment. Second, cardiometabolic risk factors were treated as binary variables and largely based on self-report, which may introduce misclassification or recall bias. Third, the sample was highly educated, which may reduce representativeness. Notably, despite high education levels, NHB participants showed lower cognitive performance. We also did not account for other potentially influential factors such as physical activity, sleep, sensory impairments (e.g., hearing or vision loss), modifiable exposures, or treatment interventions such as hypertension and diabetes medications [53]. Including these modifiable dementia risk factors in future analyses could offer a more complete picture of cognitive aging and resilience along with mitigating disparities related to specific populations. Lastly, we treated *APOE* ε4 carriership as a binary variable (≥ 1 ε4 allele vs. none), a common approach that assumes equivalent risk for heterozygotes and homozygotes. However, ε4 homozygotes (2.5%) and ε2/ε4 heterozygotes (2.0%) were rare in our sample, precluding reliable dose–response analyses. Future studies with larger numbers of ε4 homozygotes should examine potential allele‐dose effects.

This study offers several important strengths. First, the sample included predominantly cognitively unimpaired individuals (> 70%), enabling the investigation of early-stage cognitive changes prior to clinical impairment. Second, the use of a geographically localized cohort enhances internal consistency and interpretability which has been a limitation of other large community based cohorts with sites across the United States. Third, the cohort’s racial and ethnic composition allowed for stratified PIM analyses of cardiometabolic risk factors, providing valuable insights into how these exposures relate to cognitive outcomes across NHW, NHB, and Hispanic participants. By evaluating multiple cognitive domains, we were able to assess domain-specific vulnerabilities, which is crucial for designing targeted interventions given that AD pathology is related to memory changes while vascular dysfunction is associated with executive function. Fourth, a major strength lies in our use of racial ethnic stratified PIM enabling us to estimate the potential cognitive benefits of eliminating specific risk factors within each racial ethnic group. PIM combines the prevalence of the risk factor with the magnitude of association and facilitated us to estimate the potential effect of elimination of the risk factor on cognitive outcomes. While we did not model biologic susceptibility, the greater prevalence of cardiometabolic risk factors among NHB and Hispanic participants translated into larger predicted cognitive gains from risk factor elimination. This illustrates how disparities in exposure alone, regardless of interaction, can drive meaningful differences in population-level cognitive outcomes.

This study is the first to utilize population intervention models to estimate the potential benefit of removing diabetes, hypertension, dyslipidemia, obesity, and tobacco dependence across multiple cognitive domains among NHW, NHB, and Hispanic participants from a community-based cohort. These findings highlight the importance of considering both the prevalence of a risk factor as well as the strength of the association with cognitive outcomes, when evaluating racial ethnic differences. Our analyses yield counterfactual estimates of the cognitive change that might follow elimination of modifiable cardiometabolic and behavioral risk factors. While these results illustrate possible gains if risk factors were removed, they should be viewed in the context of HABS-HD cohort. Future work should include more geographically diverse participants to confirm the broader external validity of these estimates. Our findings contribute to the precision public health literature by demonstrating that tailored, racial ethnic-conscious prevention strategies may be essential for mitigating disparities in late-life cognitive decline and dementia risk. Future research should incorporate a broader range of social determinants of health, longitudinal assessments, and genetic diversity related to genotypes to further elucidate the mechanisms driving these disparities and to inform successful public health strategies for dementia prevention for all populations.

## Supplementary Information

Supplementary Material 1.

## Data Availability

The HABS-HD data is available upon request at: https://apps.unthsc.edu/itr/

## Abbreviations

AD: Alzheimer’s Disease
ADRD: Alzheimer’s Disease related Dementias
APOE: Apolipoprotein E
BMI: Body Mass Index
CI: Confidence Interval
HABS-HD: Health and Aging Brain Study Health Disparities
HbA1c: Hemoglobin A1c
MMSE: Mini-Mental State Examination
NHW: Non-Hispanic White
NHB: Non-Hispanic Black
NIA: National Institute on Aging
PIM: Population Intervention Model
SEVLT: Spanish-English Verbal Learning Test
SNP: Single Nucleotide Polymorphism
US: United States
WMS-III: Wechsler Memory Scale Third Edition
