# Supplementary material for: Population intervention models of racial ethnic disparities in cognitive outcomes from cardiometabolic risk factors – HABS-HD
Source: Alzheimers Res Ther. 2025 Oct 1;17:217. doi: 10.1186/s13195-025-01866-9 (PMC12487193; doi:10.1186/s13195-025-01866-9)
Supplement: Supplementary file 1 — Supplementary Material 1. [file 13195_2025_1866_MOESM1_ESM.docx]

| **Supplemental Table 1:** Counterfactual PIM Estimates for Eliminating Cardiometabolic Risk Factors on Harmonized Cognitive Domains, by Racial Ethnicity | | | | | | | | | | | | |
| --- | --- | --- | --- | --- | --- | --- | --- | --- | --- | --- | --- | --- |
|  | Episodic Memory  PIM (95% CI) | | | Executive Function  PIM (95% CI) | | | Processing Speed  PIM (95% CI) | | | Language  PIM (95% CI) | | |
| Risk Factor PIM: Elimination vs. Observed | NHW | NHB | Hispanic | NHW | NHB | Hispanic | NHW | NHB | Hispanic | NHW | NHB | Hispanic |
| Diabetes | NA | NA | NA | NA | NA | NA | 0.01 (0.004 – 0.02) | 0.02 (0.01 – 0.04) | 0.03 (0.01 – 0.06) | 0.01 (0.003 – 0.02) | 0.02 (0.01 – 0.04) | 0.03 (0.01 – 0.05) |
| Hypertension | NA | NA | NA | 0.03 (0.001 – 0.06) | 0.04 (0.002 – 0.08) | 0.03 (0.001 – 0.07) | NA | NA | NA | 0.04 (0.01 – 0.08) | 0.06 (0.01 – 0.11) | 0.05 (0.01 – 0.08) |
| Dyslipidemia | NA | NA | NA | NA | NA | NA | NA | NA | NA | NA | NA | NA |
| Obesity | -0.04 (-0.06 – -0.02) | -0.05 (-0.08 – -0.02) | -0.05 (-0.07 – -0.02) | NA | NA | NA | -0.03 (-0.05 – -0.01) | -0.05 (-0.08 – -0.01) | -0.04 (-0.06 – -0.01) | NA | NA | NA |
| Tobacco Dependency | 0.01(0.004 – 0.01) | 0.03 (0.01 – 0.04) | 0.02 (0.01 – 0.03) | 0.004 (0.001 – 0.01) | 0.01 (0.004 – 0.03) | 0.01 (0.002 – 0.02) | NA | NA | NA | 0.01 (0.001 – 0.01) | 0.02 (0.003 – 0.03) | 0.01 (0.002 – 0.02) |
| This table presents the population intervention model (PIM) estimates and 95% CI for the impact of non-treatment versus observed exposure patterns for hypertension, diabetes, obesity, dyslipidemia, and tobacco dependency on cognitive outcomes. PIM represent the estimated mean difference in cognitive performance across four domains—memory, executive function, processing speed, and language—for Non-Hispanic White, Non-Hispanic Black, and Hispanic participants. A **positive PIM** means that the average outcome under the “no-exposure” (non-treatment) scenario is **higher** than the observed mean—i.e. eliminating the risk factor would **improve** the outcome. A **negative PIM** means that the average outcome under the “no-exposure” scenario is **lower** than the observed mean—i.e. eliminating the risk factor would be associated with a **worse** outcome compared to what is observed in the sample. The results highlight disparities in the impact of these risk factors on cognitive outcomes between racial groups. Abbreviation: NHW Non-Hispanic White, PIM population intervention model, CI confidence interval | | | | | | | | | | | | |
